# Analysis of partial weight bearing after surgical treatment in patients with injuries of the lower extremity

**DOI:** 10.1007/s00402-020-03588-z

**Published:** 2020-09-03

**Authors:** Alexander Maximilian Eickhoff, Raffael Cintean, Carina Fiedler, Florian Gebhard, Konrad Schütze, Peter H. Richter

**Affiliations:** Department of Traumatology, Hand-, Plastic-, and Reconstructive Surgery, Center of Surgery, University of Ulm, Albert- Einstein- Allee 23, 89081 Ulm, Germany

**Keywords:** Partial weight bearing, Loading, Electronic shoe soles

## Abstract

**Introduction:**

After surgical treatment of injuries of the lower extremity, partial weight bearing is often suggested until soft tissue consolidation. It is doubtful, if this recommendation can be implemented, even in the case that a patient is performing partial weight bearing with a physical therapist. Consequently the question remains, if patients are able to implement partial weight bearing after surgery and which factors favor incompliance.

**Materials and methods:**

49 patients, who underwent surgical treatment after injuries of the lower extremity, were equipped with electronic shoe insoles on both sides. Different weight bearing instructions were given depending on the type of injury and surgery (full weight bearing vs. 20 kg weight bearing vs. non-weight bearing). Besides loading, other factors like age, gender, weight and physical activity were evaluated. Statistical analysis was performed using Chi-square and Fisher’s exact test with significance set at a *p* value < 0.05.

**Results:**

25 of the 40 patients, who had to perform non- or partial weight bearing, were not able to follow postoperative instructions (compliance rate 37.5%). The average loading of the whole collective was 32.6 kg (4.8–109.2 kg). The specification of loading had no statistically significant influence on real loading (*p*-value 0.39). Elderly patients were less able to follow instructions than younger patients (36 vs 30.2 kg). Physically active compared to non-active patients overloaded their injured extremity (37.8 vs 28.7 kg). Patients with a high body mass index (BMI) encountered more difficulties to perform partial weight bearing than lightweight patients (36.9 vs 25.1 kg).

**Conclusions:**

Most patients were not able to follow loading limitation, even a few days after surgery and even if the patients were trained by a physiotherapist. Excessive weight bearing-related complications should be evaluated.

## Introduction

Partial or even non-weight bearing is often recommended by trauma surgeons after lower extremity surgery. Aim of partial weight bearing is to create optimal requirements for good bone and soft tissue healing and to reduce implant failures [1]. In addition, early full weight bearing can be associated with secondary fracture dislocation [2]. Yet, a steady increase in weight bearing produces not only a faster bone healing, but also a better quality of the newly formed tissue [2]. Avoidance of weight bearing furthermore favors the emergence of deep leg vein thrombosis. Partial weight bearing of 20 kg leads to nearly the same venous return current as full weight bearing [3].

Teaching or visualization of the correct postoperative load remains a problem even if patients were instructed by physiotherapists. A common method to visualize the weight is with the help of scales, although many studies did not show any benefit concerning patient compliance [4–6].

Moreover, the use of crutches and a wheeled-walker is often badly taught. Besides the disability of performing partial weight bearing complications like abrasions of the skin and nerve damages can be observed, especially when using armpit crutches [7, 8]. Many different walking techniques are known for crutches, depending on the injury and the existence of other disabilities. Three-point crutch gait, creating three contact points at the same time, seemed to be favorable, if partial weight bearing after surgery of a lower extremity is required [8].

Use of wheeled-walker or mobilization with only one cane is not suitable for partial weight bearing [9].

Two different methods are available for analysis of the load. The force platform is the gold standard, because it is the most valid method [10]. However, only a snapshot can be identified and incorrect data can easily be acquired. Another possibility is the use of electronic shoe insoles.

Requirements to an optimal system should include the potential to record and storage data for a few days. On the other hand, it should be easy to use and the patient should not notice that each step is registered. Therefore, the moticon OpenGo (Moticon ReGo AG) system was chosen for this study.

A high potential for measurement of force parameters during the mobilization compared to the AMTI force-plate system and other electronic systems, could be demonstrated [11].

Nowadays accelerated rehabilitation is very popular to achieve early mobilization and not at least to shorten the length of stay [1].

The hypothesis of this study was that especially old and multimorbid patients are not able to follow surgeons partial weight bearing instructions. On the other hand, young patients, who are physical active, should be able to perform partial weight bearing especially, if they are practicing with a physiotherapist. Another purpose was to investigate, if pain was negatively correlated with weight bearing, which was observed in other studies [12].

The aim of this examination is furthermore to determine factors favoring incorrect loading.

## Materials and methods

In this prospective study, 61 patients were equipped with electronic shoe insoles. 49 patients, who underwent surgical treatment after injuries of the lower extremity in a Level I trauma center, were included in this study. Assignment of the patients was done randomly.

The shoes of each proband were equipped with special electronic shoe insoles on the first day after surgery. The insoles include 13 sensors, which are able to perform measurement of pressure, balance and movement.

Data could be downloaded wireless or via USB. Analysis was executed with a software (Moticon Beaker^®^), which is able to generate different reports, giving information about the average of maximum loading.

The insoles also have a smart record function to save battery and memory capacity.

Patients with dementia, combined injuries of both legs or under 18 years of age were excluded.

Furthermore patients with comorbidities, which may influence the ability to use crutches, like hemiplegia or disabilities of the upper extremities were excluded. There were no restrictions regarding the type of fracture/injury of the lower leg leading to a variety of included injuries and surgical procedures (e.g., nail osteosynthesis of pertrochanteric fracture or ACL replacement).

Measurement lasted 24 to 101 h, depending on the time of discharge and intensity of movement. Every patient was instructed by a physiotherapist at least once a day using bathroom scales to meet weight bearing instructions. Each patient was trained to perform a 3-point crutch gait, knowing that users could reproduce partial weight bearing more accurately compared to a two-point or a four-point gait.

To discover factors influencing the load many other factors like age, gender, weight, medication, pain and physical activity were evaluated by including patients in the study. The Numerical Rating Scale (NRS) was used to receive a sufficient pain assessment.

Incompliance was defined as exceeding the limitation of 20 kg.

Statistical analysis was performed after consultation of our department for statistics using the Chi-square, the *t*-test and the Fisher’s exact test with significance set at a *p* value < 0.05.

## Results

61 patients were equipped with electronic shoe insoles. 49 cases could be used for statistical analysis. Consequently, a dropout rate of 20% (12 probands) was observed, explained by technical problems of the shoe insoles. Mean age was 56.3 years (range 19–92 years). Partial weight bearing of 20 kg was recommended in 39 patients. 9 persons were allowed to perform full weight bearing and 1 patient was not allowed to bear any weight on the injured leg. Average load of the whole collective was 32.6 kg (4.8–109.2 kg). 15 patients with load limitation were able to follow the instructions. Consequently, 25 patients could not meet partial weight bearing instructions (compliance rate 37.5%). Average load of the patients, which were supposed to perform partial weight bearing, was 27.3 kg. Nevertheless, no statistical significant correlation between the postoperative behavior instructions and the real loading could be seen (*p*-value 0.39).

**Fig. 1:**
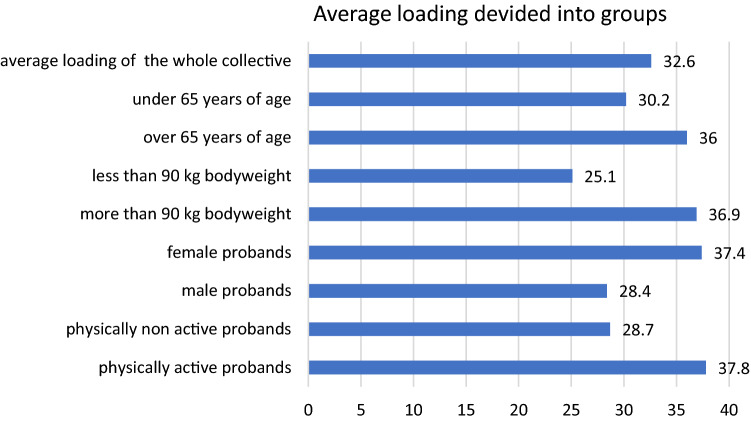
Average loading in kilogram divided into different groups

Elderly patients (> 65 years of age) encountered bigger difficulties to obey instructions compared to younger patients (36 vs. 30.2 kg, *p*-value 0.27) even though their body weight was lower (73.8 vs. 77.1 kg). Active patients, who performed physical exercise at least once a week prior to surgery, overloaded their injured extremity more than non-active patients (37.8 kg vs 28.7 kg, *p*-value 0.79). Female patients bore more weight on the operated extremity than male patients (37.4 vs. 28.4 kg, *p*-value 0.32). Patients with a body mass index (BMI) over 25 followed instructions worse than lightweight patients (36.9 vs 25.1 kg, *p*-value 0.58, Fig. 1).

Subjective pain evaluation, measured by the Numerical Rating Scale (NRS), seemed to have an influence on weight bearing. Patients with a score of 1–5 showed loading of 34.8 kg, whereas patients with NRS of 6–10 bore only 25.2 kg on the injured leg. However, there was no statistic significant influence on incorrect loading (*p* value 0.08).

In general, loading of the healthy side was much higher compared to the injured side (79.1 kg vs. 32.6 kg, *p* value 0.01). Existence of comorbidities did not influence the loading (*p*-value 0.325).

Standing time on the healthy side was noticeably higher compared to the injured side (72.9% vs. 63.3%). In addition, the gaitline was shorter on the injured side (88.6 mm vs. 134.4 mm).

## Discussion

The purpose of partial weight bearing after orthopedic trauma surgery has been discussed for many years. Supporters believe that early full weight bearing can favor malunion and implant failure [13]. Moreover, steady increase of weight results in faster bone healing and a better quality of newly formed tissue [2].

On the other hand, multiple studies showed difficulties in partial weight bearing after surgery of the lower extremity [14–16]. Even healthy probands were not able to properly follow weight bearing instructions [14]. Braun et al. monitored 30 patients with Weber B, tibia shaft and intertrochanteric fractures. They demonstrated that 53% of the patients were not compliant [15]. Chiodo et al. observed 51 patients, who had been instructed to perform non-weight bearing for more than 3 weeks and discovered that in 27.5% instructions could not be followed [16]. These differences can be explained by the study design. Chiodo et al. used pressure-sensitive films, whereas Braun et al. equipped the probands with electronic shoe insoles. Also the definition of non-compliance was different. Chiodo defined the maximum detectable pressure exerted in more than 50% of the film as a non-compliance. Braun et al. determined non-compliance in case of achieving the weight bearing recommendation in less than 30%. Inclusion criteria were different as well. Whereas Chiodo et al. included patients with “unilateral lower extremity abnormalities”, Braun et al. chose patients with ankle, tibial shaft and intertrochanteric fractures. We observed a non-compliance rate of even 62.5% in this study. This high number can be explained by the definition of compliance. To identify the exact weight bearing of the patients, a special parameter called “the average of the maximum loading of all steps” was implied in the moticon beaker^®^ software, which was defined as maximum loading of each step and calculation of the average of all steps. If the average of loading was higher than the recommendation of 20 kg it was defined as non-compliance, knowing that the system tends to show a lower load [17]. The reason for this study design was to get an exact impression of the number of non-compliance and severity of heavy weight bearing in the patient collective. This study also showed that postoperative behavior instructions had no statistically significant influence on the real loading (*p*-value 0.39). There was a significant difference between loading of the injured (32.6 kg) and the not-injured leg (79.1 kg, *p* value 0.1). It can be concluded that the patient himself reduces the load of the injured extremity independent of recommendations made by the surgeon. Therefore, the Numerical Rating Scale (NRS) was used to objectify the pain. A trend to a lower loading in case of an increase in pain could be seen (*p* value 0.08).

Hurkmans et al. found out that female gender and walking time seemed to be positively associated with excessive loading. On the other hand, pain had a negative effect.

General experience showed that compliance of patients decreases, if a lower weight bearing limit is defined and if the patient is walking at home without observation of a physical therapist [12].

These findings are in line with our results. We could also show that female probands bear much more weight on the surgically treated leg compared to male probands (37.4 vs. 28.4 kg). Furthermore, pain seemed to have an influence on weight bearing without showing any significance.

Ruckstuhl et al. compared the ability to perform partial weight bearing with psychomotoric skills. Matrix insoles and the so-called “Motorische Leistungsserie (MLS)” were used. Their conclusion was that weight and age of patients were in line with the loading. It implies that heavier and older people were less able to follow weight bearing restrictions. Patients, who were able to perform partial weight bearing, showed significant better psychomotoric skills in the MLS subtests [18].

Comparable results could be detected in our study. Elderly patients, more or less expected, were not able to follow instructions of the surgeon properly, even if the workout was performed together with a physiotherapist, who tried to visualize the loading with a scale. Average loading of the elderly patients with loading restriction of 20 kg was 36 kg. Only 4 of the 16 elderly patients met the loading limitation (compliance rate 25%) resulting in a reduced compliance rate compared to the whole collective (compliance rate of 37.5%).

Also Patients with a BMI over 25 followed instructions worse than lightweight patients (36.9 vs 25.1 kg), without showing a statistical significance (*p*-value 0.58).

Surprisingly, even young patients were not able to perform partial weight bearing. Average loading was 30.2 kg and only 11 of 26 patients followed the weight bearing limit (compliance rate 42%).

Different methods can be used, to improve the ability to perform partial weight bearing. All patients were instructed by a physiotherapist during the hospital stay on how to achieve the loading limitation. Scales are typically used to visualize the loading of 20 kg, which was also applied in this investigation. Different studies were performed to evaluate the best way to achieve patient compliance. A review of Hustedt et al. demonstrated that biofeedback training seemed to be the best method to achieve partial weight bearing, especially compared to training with bathroom scales, which seems to be inferior [4, 19]. In most of these studies audio feedback and not haptic feedback was used. It remains unclear, if haptic biofeedback is even better than audio feedback [4]. Our aim was to investigate, if patients were able to follow partial weight bearing instructions in an everyday scenario, where most of the patients do not have the possibility to use a biofeedback system. This is the reason, why biofeedback was not part of this study.

The purpose of partial weight bearing is still discussed. Training with a biofeedback system would be useful, if complications could be avoided. Therefore further investigations are mandatory to analyze, if incorrect loading favors the number of complications. If a correlation can be demonstrated, we should determine new training methods to improve the compliance of patients. If no correlation can be detected, the purpose of partial weight bearing should be challenged.

A limitation of this study is the small number of cases in combination with the big number of study dropouts due to malfunctional soles. Especially the so-called smart record function, which allows to record only in case of movement to save battery, did not work faultlessly.

Besides, the inhomogeneity of the included patients with high number of different injuries leads to limitations for particular fractures and surgical procedures. Furthermore, the limited follow up period and short time of measurement reduces the meaningfulness of the results.

In addition, complications as a result of immobilization or excessive weight bearing were not mentioned in this study, knowing that especially in elderly patients immobilization may increase the risk of muscle atrophies and the number of pneumonia [20].

Furthermore, Tian et al. and de Boer et al. agreed with the statement that there is not any influence between an early weight bearing and the occurence of complications [21, 22].

## Conclusions

Electronic shoe insoles are a good tool to observe the postoperative weight bearing behavior of patients. This study shows that especially elderly patients were not able to follow the surgeons weight bearing instructions. Further investigations should be performed to analyze the correlation between excessive weight bearing and the occurrence of complications. If no correlation can be detected, we should rethink the purpose of weight bearing instructions.
